# Decreased factor XIII activity and haptoglobin may be markers of enhanced-fibrinolytic-type disseminated intravascular coagulation status: Case report

**DOI:** 10.1097/MD.0000000000043123

**Published:** 2025-07-25

**Authors:** Shinya Yamada, Hideyasu Ueda, Yukio Suga, Toshihiro Miyamoto, Hidesaku Asakura

**Affiliations:** a Department of Hematology, Kanazawa University Hospital, Kanazawa, Japan; b Department of Cardiovascular Surgery, Kanazawa University Hospital, Kanazawa, Japan; c Faculty of Pharmacy, Institute of Medical, Pharmaceutical and Health Science, Kanazawa University, Kanazawa, Japan.

**Keywords:** aortic aneurysm, disseminated intravascular coagulation, enhanced-fibrinolytic-type DIC, factor XIII, haptoglobins

## Abstract

**Rationale:**

Aortic aneurysms are often accompanied by enhanced-fibrinolytic-type disseminated intravascular coagulation (DIC), but markers to adequately assess DIC status remain lacking.

**Patient concerns:**

An 80-year-old man scheduled for thoracic endovascular aortic repair for aortic aneurysm was diagnosed with enhanced-fibrinolytic-type DIC. The concern was whether surgery could be safely performed in a state of enhanced-fibrinolytic-type DIC with a bleeding tendency.

**Interventions:**

He was treated with a combination of heparin and tranexamic acid.

**Outcomes:**

DIC was well controlled and the surgery was performed safely. However, DIC worsened again postoperatively. Since no severe bleeding symptoms were observed, no therapeutic intervention for DIC was performed postoperatively. During this period, coagulation tests including factor XIII activity and haptoglobin were followed over time to clarify the relevance of these markers.

Factor XIII activity was decreased and haptoglobin was mildly decreased in DIC. Improvement of DIC led to normalization of both markers. Re-exacerbation of DIC led to decreased factor XIII activity and mildly decreased haptoglobin.

**Lessons:**

Factor XIII activity and haptoglobin appear to represent useful markers of DIC status.

## 1. Introduction

Disseminated intravascular coagulation (DIC) is a severe condition in which systemic and persistently marked activation of coagulation occurs in the presence of underlying disease.^[1]^ DIC is categorized as a thrombosis with thrombocytopenia syndrome because of the presentation with systemic microthrombosis and consumptive thrombocytopenia due to marked activation of coagulation.^[2]^ DIC is associated with activation of fibrinolysis as well as coagulation, but the degree of fibrinolytic activation varies considerably depending on the underlying disease.^[1]^ DIC with marked activation of fibrinolysis and bleeding symptoms is called enhanced-fibrinolytic-type DIC.^[1]^ Aortic aneurysms are often the underlying cause of enhanced fibrinolytic-type DIC. The results of coagulation tests in enhanced-fibrinolytic-type DIC associated with aortic aneurysm are characterized by low platelet counts, normal to prolonged prothrombin time (PT), mildly shortened to prolonged activated partial thromboplastin time (APTT), and markedly decreased fibrinogen, markedly increased fibrin/fibrinogen degradation products (FDP), increased D-dimer, a high FDP-to-D-dimer ratio, markedly increased thrombin-antithrombin complex (TAT) as a marker of coagulation activation, markedly increased plasmin-α_2_ plasmin inhibitor complex (PIC) as a marker of fibrinolytic activation, markedly decreased α_2_ plasmin inhibitor (α_2_ PI), and decreased plasminogen.^[1,3,4]^

In DIC, factor XIII activity is often markedly decreased^[5,6]^ and haptoglobin shows variable behavior ranging from mildly decreased to increased.^[6]^ On the other hand, while thrombotic microangiopathy (TMA) is categorized as a thrombosis with thrombocytopenia syndrome like DIC and causes consumptive thrombocytopenia due to systemic and persistent platelet activation, factor XIII activity is normal, but haptoglobin is significantly decreased. These behaviors differ markedly from DIC.^[6]^

No previous reports appear to have examined the time course of factor XIII activity and haptoglobin with disease control of DIC in cases with therapeutic intervention for DIC. The present study examined coagulation markers (including factor XIII activity) and haptoglobin to clarify the relationships between the disease status of DIC and factor XIII activity and haptoglobin, revealing some interesting findings.

## 2. Case report

An 80-year-old Japanese man had undergone arch replacement for Stanford type A aortic dissection 2 years earlier. This time, he was scheduled to undergo thoracic endovascular aortic repair (TEVAR) for a worsening thoracic aortic aneurysm. Scattered purpura were observed throughout the body.

Laboratory tests showed: platelet count, 79,000/μL (reference: 158,000–348,000/μL); PT, 12.6 seconds (reference: 10.5–13.0 s); APTT, 35.2 seconds (reference: 24.0–34.0 s); fibrinogen, 111 mg/dL (reference: 181–378 mg/dL); FDP, 147.4 μg/mL (reference: < 5.0 μg/mL); D-dimer, 42.3 μg/mL (reference: ≤ 1.0 μg/mL); TAT, 60.1 ng/mL (reference: < 4 ng/mL); PIC, 8.5 μg/mL (reference: < 1.1 μg/mL); α_2_ PI, 32% (reference: 70–130%); and plasminogen, 58% (reference: 70–130%).

The patient showed a score of 7 (≥6 points for DIC diagnosis) according to the Japanese Society on Thrombosis and Hemostasis DIC diagnostic criteria^[7]^ and met the diagnostic criteria for DIC. Based on the values of PIC, α_2_ PI, FDP, and fibrinogen, the patient was considered to show enhanced-fibrinolytic-type DIC.^[1]^ At this time, factor XIII activity was 24% (reference: 70–130%) and haptoglobin was 12 mg/dL (reference: 43–180 mg/dL).

**Figure 1.**
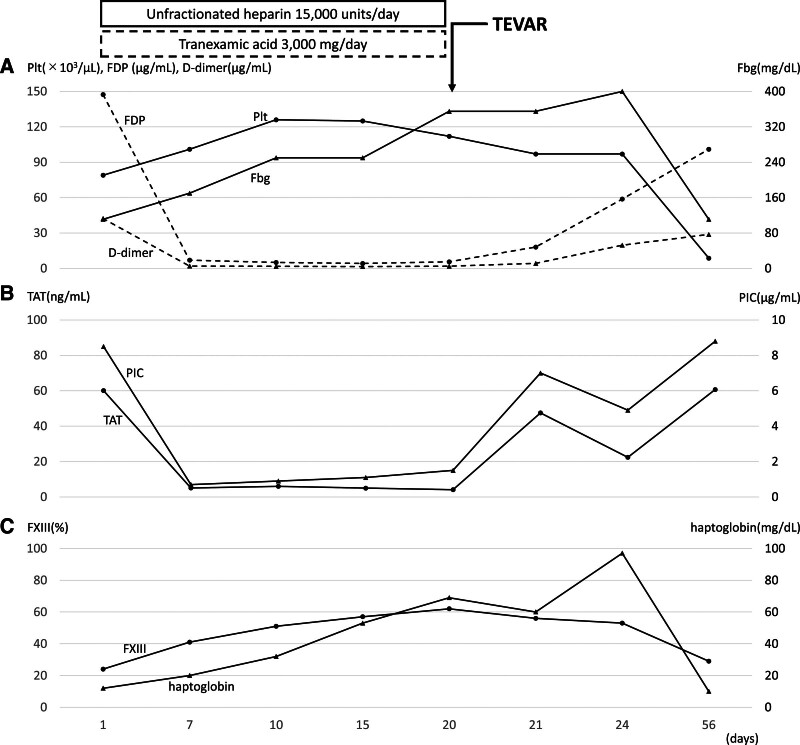
Time course of DIC markers and factor XIII activity and haptoglobin TEVAR was performed on day 20 after initiation of heparin/tranexamic acid combination therapy for DIC. Postoperative DIC therapy was not resumed and the patient was discharged 8 days after surgery. At an outpatient visit 36 days after surgery (56 days after starting heparin/tranexamic acid combination therapy), scattered purpura were observed all over the body. (A) Changes in platelet number, FDP, D-dimer and fibrinogen. The solid line marked with (•) indicates platelet count, the solid line marked with (▲) indicates fibrinogen, the dotted line marked with (•) indicates FDP, and the dotted line marked with (▲) indicates D-dimer. With the start of therapeutic intervention for DIC, platelet count and fibrinogen recovered and FDP and D-dimer decreased. However, 56 days after initiation of heparin/tranexamic acid combination therapy, platelet count decreased, fibrinogen decreased, and FDP and D-dimer increased. (B) Changes in TAT and PIC. (•) indicates TAT as a coagulation marker and (▲) indicates PIC as a fibrinolytic marker. With the start of therapeutic intervention for DIC, TAT and PIC decreased markedly. However, 56 days after initiation of heparin/tranexamic acid combination therapy, TAT and PIC were elevated again. Based on the results of (A) and (B), DIC was controlled during heparin/tranexamic acid combination therapy, but remained after TEVAR, and re-aggravation of coagulation/fibrinolytic markers was considered to have occurred. (C) Changes in factor XIII activity and haptoglobin. (•) indicates factor XIII activity and (▲) indicates haptoglobin. Both factor XIII activity and haptoglobin improved while DIC was under control, but both factor XIII activity and haptoglobin decreased when DIC re-exacerbated. DIC = disseminated intravascular coagulation, TEVAR = thoracic endovascular aortic repair, Plt = platelets, FDP = fibrin/fibrinogen degradation products, Fbg = fibrinogen, TAT = thrombin-antithrombin complex, PIC = plasmin-α_2_ plasmin inhibitor complex.

To control bleeding and ensure safety during surgery, DIC needed to be controlled prior to surgery. Continuous infusion of unfractionated heparin was initiated at 15,000 units/24 h in combination with tranexamic acid at 3 g/24 h.^[3,4,8]^ The course of treatment is shown in Figure 1. On day 7 of combination therapy with heparin and tranexamic acid, platelet count and fibrinogen were recovered, FDP and D-dimer were decreased, and both TAT and PIC were markedly decreased, and the patient was recovered from enhanced-fibrinolytic-type DIC. In addition, the scattered purpura disappeared. Finally, coagulation tests showed improvement of DIC, with: platelet count, 112,000/μL; PT, 12.6 seconds; APTT, 42.7 seconds; fibrinogen, 355 mg/dL; FDP, 5.6 μg/mL; D-dimer, 1.9 μg/mL; TAT, 4.1 ng/mL; PIC, 1.5 μg/mL; α_2_ PI, 91%; and plasminogen, 50%. Continuous infusion of heparin/tranexamic acid combination therapy was finished 4 hours before surgery and TEVAR was performed. Intraoperative bleeding was slight and the surgery was performed safely. Immediately before surgery, factor XIII activity was 62% and haptoglobin was 69 mg/dL. Postoperatively, there was no wound bleeding and the patient was discharged on postoperative day 8 without resuming continuous infusion of heparin/tranexamic acid combination therapy.

At an outpatient visit 36 days after surgery, scattered purpura was again identified. Coagulation tests at this time showed recurrent enhanced-fibrinolytic-type DIC: platelet count, 87,000/μL; PT, 12.8 seconds; APTT, 32.0 seconds; fibrinogen, 111 mg/dL; FDP, 101.0 μg/mL; D-dimer, 28.8 μg/mL; TAT, 60.7 ng/mL; PIC, 8.8 μg/mL; α_2_ PI, 66%; and plasminogen, 95%. Factor XIII activity was 29% and haptoglobin was 10 mg/dL. As no severe bleeding was observed, according to the clinical practice guideline for DIC,^[8]^ therapeutic intervention was withheld, and the patient was closely monitored. Throughout follow-up, no increases were seen in lactate dehydrogenase or indirect bilirubin levels and no schistocytes were identified.

## 3. Discussion

In this report, a decrease in factor XIII activity and haptoglobin was observed when DIC was first diagnosed. With the improvement of DIC under combination therapy with heparin and tranexamic acid, factor XIII activity and haptoglobin were normalized. However, factor XIII activity and haptoglobin decreased again with the re-exacerbation of DIC. This appears to be the first report in the world to follow the behaviors of factor XIII activity and haptoglobin in relation to the disease status of DIC, and showed that the decreases in factor XIII activity and haptoglobin could be used as markers indicative of DIC progression.

Factor XIII is thought to be consumptively decreased due to excessive activation of coagulation in DIC. The effectiveness of factor XIII replacement as a means of addressing bleeding symptoms in enhanced fibrinolytic-type DIC has also been reported.^[9,10]^

In DIC, the marked activation of coagulation leads to the formation of multiple fibrin microthrombi, which in turn leads to mechanical hemolysis and a decrease in haptoglobin levels. TMA similarly causes thrombus formation and consequent mechanical hemolysis, although the primary site of lesions in both DIC and TMA is at the level of microcirculation. The degrees of hemolysis are considered to differ because thrombus formation is on the slow-flowing venous side in DIC, but on the fast-flowing arterial side in TMA.^[11]^ The decrease in haptoglobin is thus marked in TMA, but only mild in DIC.^[6]^ Further, since haptoglobin is an inflammatory protein,^[12–14]^ production is increased in septic DIC, and haptoglobin may exhibit a variety of behaviors from mildly decreased to increased. In enhanced-fibrinolytic-type DIC due to aortic aneurysm, the microthrombi formed are lysed one after another by excessive fibrinolytic activation, so mechanical hemolysis is only mild and haptoglobin is considered mildly decreased to normal. When haptoglobin is reduced, the hemoglobin, heme, and Fe^2+^ produced from hemolysis are not processed and become active as damage-associated molecular patterns. This exacerbation of inflammation and free radical formation may contribute to organ damage.^[15]^ Since the haptoglobin reduction seen in sepsis has been reported as an exacerbating factor for organ damage^[16]^ and poor prognosis,^[17]^ whether haptoglobin replacement is effective in preventing organ damage among DIC patients with reduced haptoglobin represents an important issue to clarify in future investigations.

Several limitations to this report need to be kept in mind when interpreting the results. The first is regarding the diagnosis of DIC. This case did not meet the diagnostic criteria for DIC as set out by the International Society on Thrombosis and Haemostasis.^[18]^ This is because the International Society on Thrombosis and Haemostasis DIC diagnostic criteria were originally developed for septic DIC^[18]^ and are not sufficiently sensitive for enhanced-fibrinolytic-type DIC.^[1]^ In this study, the Japanese Society on Thrombosis and Hemostasis DIC diagnostic criteria were used.^[7]^ Second, the findings in this study were based on a single case, and accumulation of further cases is thus needed. Third, factors other than DIC can affect haptoglobin levels. In this case, TEVAR was performed on day 20 of combination therapy with heparin/tranexamic acid. One possibility is that postoperative haptoglobin production was increased due to the invasiveness and inflammation of the surgery, and the haptoglobin level on day 24 may have been affected by this.

Until now, hemolysis in DIC has received scant attention, possibly because it has been observed only to the extent that hemolysis is not reflected in lactate dehydrogenase or indirect bilirubin levels. However, hemolysis occurs in DIC, and the associated release of damage-associated molecular patterns may contribute to organ damage. Monitoring hemolysis by haptoglobin is therefore also important in DIC.

In the future, we hope to examine variations in factor XIII and haptoglobin in DIC for each underlying disease.

## 4. Conclusion

We have reported an association between the status of enhanced-fibrinolytic-type DIC associated with aortic aneurysm, bleeding symptoms, factor XIII activity, and haptoglobin. Decreased factor XIII activity and haptoglobin may offer useful markers of disease progression in enhanced fibrinolytic-type DIC. In addition, the measurement of these markers may provide critical insights into the complex pathophysiology of enhanced-fibrinolytic-type DIC, potentially leading to the development of novel DIC treatments in the future. Further research, including large-scale prospective studies and mechanistic investigations, is warranted to validate the clinical utility of factor XIII and haptoglobin as diagnostic and prognostic biomarkers and to explore their potential as therapeutic targets in this challenging clinical entity.

## Acknowledgments

We would like to thank FORTE Science Communications (www.forte-science.co.jp) for English language editing.

## Author contributions

**Conceptualization:** Shinya Yamada, Hidesaku Asakura.

**Supervision:** Hideyasu Ueda, Yukio Suga.

**Validation:** Shinya Yamada.

**Visualization:** Shinya Yamada.

**Writing – original draft:** Shinya Yamada.

**Writing – review & editing:** Shinya Yamada, Hideyasu Ueda, Yukio Suga, Toshihiro Miyamoto, Hidesaku Asakura.
